# Supplementary material for: Farm-to-fork changes in poultry microbiomes and resistomes in Maputo City, Mozambique
Source: mSystems. 2024 Dec 19;10(1):e01037-24. doi: 10.1128/msystems.01037-24 (PMC11748491; doi:10.1128/msystems.01037-24)
Supplement: Supplemental material — Supplemental table and figures. [file msystems.01037-24-s0001.docx]

**Farm-to-fork Changes in Poultry Microbiomes and Resistomes in Maputo City, Mozambique**

Natalie Olson^1^, Frederica Lamar^1^, Hermógenes Mucache^2^, José Fafetine^2^, Joaquim Saíde**^3^**, Amelia Milisse**^3^**, Denise Brito**^3^**, Kelsey J**.** Jesser**^4^**, Karen Levy**^4^**, Matthew Freeman^1^, Maya L. Nadimpalli^1#^

**Appendix**

| **Tables** | **Page** |
| --- | --- |
| Table S1: Sample accession numbers for 101 chicken fecal, carcass, and rinse bucket metagenomes along the poultry value chain in Mozambique, BioProject number PRJNA1140288  Table S2: Metadata for all samples available for sequencing, including those with insufficient DNA for sequencing  Table S3: High-Risk Antimicrobial Resistance Genes Ranking in the First Quartile of Zhang et al. 2022 | 1-3 |
|  |  |
| **Figures** |  |
| Figure S1-S4: Summaries of bacteria, pathogens, ARGs, and HR-ARGs detected in fecal metagenomes from poultry production sites (farms or households). | 4-7 |
| Figure S5-8S: Summaries of bacteria, pathogens, ARGs, and HR-ARGs detected in fecal metagenomes from market sites. | 8-11 |
| Figure S9-11: Summaries of bacteria, pathogens, ARGs, and HR-ARGs detected in poultry carcass metagenomes from market sites | 12-14 |
| Figure S12: Stacked bar plot depicting proportional abundance of high-risk ARGs (HR-ARGs) detected in commercial carcass metagenomes from market sites. | 14 |

Table S1. Sample accession numbers for 101 chicken fecal, carcass, and rinse bucket metagenomes along the poultry value chain in Mozambique, BioProject number PRJNA1140288

| Accession | Sample Name | Location | Breed | Sample Type |
| --- | --- | --- | --- | --- |
| SAMN42828289 | A101 | Market | Commercial | Fecal |
| SAMN42828290 | A104 | Market | Commercial | Fecal |
| SAMN42828291 | A111 | Market | Commercial | Fecal |
| SAMN42828292 | A117 | Market | Local | Carcass |
| SAMN42828293 | A120 | Market | Commercial | Carcass |
| SAMN42828294 | A121 | Market | Commercial | Carcass |
| SAMN42828295 | A124 | Market | Commercial | Carcass |
| SAMN42828296 | A127 | Market | Commercial | Carcass |
| SAMN42828297 | A128 | Market | Commercial | Carcass |
| SAMN42828298 | A130 | Market | Commercial | Carcass |
| SAMN42828299 | A131 | Market | Commercial | Carcass |
| SAMN42828300 | A132 | Market | Commercial | Carcass |
| SAMN42828301 | A140 | Market | Commercial | Fecal |
| SAMN42828302 | A142 | Market | Commercial | Fecal |
| SAMN42828303 | A144 | Production (Farm) | Commercial | Fecal |
| SAMN42828304 | A147 | Market | Commercial | Carcass |
| SAMN42828305 | A148 | Market | Commercial | Carcass |
| SAMN42828306 | A150 | Market | Commercial | Carcass |
| SAMN42828307 | A151 | Market | Commercial | Carcass |
| SAMN42828308 | A154 | Market | Commercial | Carcass |
| SAMN42828309 | A155 | Market | Commercial | Carcass |
| SAMN42828310 | A158 | Market | Commercial | Fecal |
| SAMN42828311 | A161 | Market | Local | Fecal |
| SAMN42828312 | A173 | Production (Household) | Local | Fecal |
| SAMN42828313 | A177 | Production (Household) | Local | Fecal |
| SAMN42828314 | A179 | Production (Household) | Local | Fecal |
| SAMN42828315 | A184 | Production (Household) | Local | Fecal |
| SAMN42828316 | A192 | Production (Household) | Local | Fecal |
| SAMN42828317 | A193 | Market | Local | Fecal |
| SAMN42828318 | A200 | Production (Household) | Local | Fecal |
| SAMN42828319 | A203 | Production (Household) | Local | Fecal |
| SAMN42828320 | A208 | Market | Local | Fecal |
| SAMN42828321 | A209 | Market | Local | Fecal |
| SAMN42828322 | A216 | Production (Farm) | Commercial | Fecal |
| SAMN42828323 | A217 | Market | Commercial | Fecal |
| SAMN42828324 | A233 | Production (Farm) | Commercial | Fecal |
| SAMN42828325 | A234 | Production (Farm) | Commercial | Fecal |
| SAMN42828326 | A83 | Market | Local | Carcass |
| SAMN42828327 | A84 | Market | Local | Carcass |
| SAMN42828328 | A88 | Market | Local | Carcass |
| SAMN42828329 | A89 | Market | Local | Carcass |
| SAMN42828330 | A90 | Market | Local | Carcass |
| SAMN42828331 | A91 | Market | Local | Carcass |
| SAMN42828332 | A92 | Market | Local | Fecal |
| SAMN42828333 | A93 | Market | Local | Fecal |
| SAMN42828334 | A94 | Market | Local | Fecal |
| SAMN42828335 | A98 | Market | Commercial | Fecal |
| SAMN42828336 | EUP2044 | Market | Commercial | Carcass |
| SAMN42828337 | EUP2047 | Market | Commercial | Rinse Bucket |
| SAMN42828338 | EUP2049 | Market | Commercial | Rinse Bucket |
| SAMN42828339 | EUP2050 | Market | Commercial | Carcass |
| SAMN42828340 | EUP2051 | Market | Commercial | Rinse Bucket |
| SAMN42828341 | EUP2057 | Market | Commercial | Rinse Bucket |
| SAMN42828342 | EUP2058 | Market | Commercial | Carcass |
| SAMN42828343 | EUP2060 | Market | Commercial | Carcass |
| SAMN42828344 | EUP2061 | Market | Commercial | Rinse Bucket |
| SAMN42828345 | EUP2062 | Market | Commercial | Carcass |
| SAMN42828346 | EUP2063 | Market | Commercial | Rinse Bucket |
| SAMN42828347 | EUP2066 | Market | Commercial | Carcass |
| SAMN42828348 | EUP2067 | Market | Commercial | Rinse Bucket |
| SAMN42828349 | EUP2068 | Market | Commercial | Carcass |
| SAMN42828350 | EUP2093 | Market | Commercial | Rinse Bucket |
| SAMN42828351 | EUP2101 | Market | Commercial | Rinse Bucket |
| SAMN42828352 | EUP2109 | Market | Commercial | Rinse Bucket |
| SAMN42828353 | EUP2112 | Market | Commercial | Carcass |
| SAMN42828354 | EUP2113 | Market | Commercial | Rinse Bucket |
| SAMN42828355 | EUP2115 | Market | Commercial | Rinse Bucket |
| SAMN42828356 | EUP2116 | Market | Commercial | Carcass |
| SAMN42828357 | EUP2117 | Market | Commercial | Rinse Bucket |
| SAMN42828358 | EUP2118 | Market | Commercial | Carcass |
| SAMN42828359 | EUP2126 | Market | Commercial | Carcass |
| SAMN42828360 | EUP2127 | Market | Commercial | Rinse Bucket |
| SAMN42828361 | EUP2128 | Market | Commercial | Carcass |
| SAMN42828362 | EUP2129 | Market | Commercial | Rinse Bucket |
| SAMN42828363 | EUP2130 | Market | Commercial | Carcass |
| SAMN42828364 | EUP2131 | Market | Commercial | Rinse Bucket |
| SAMN42828365 | EUP2132 | Market | Commercial | Carcass |
| SAMN42828366 | EUP2133 | Market | Commercial | Rinse Bucket |
| SAMN42828367 | EUP2134 | Market | Commercial | Carcass |
| SAMN42828368 | EUP2135 | Market | Commercial | Rinse Bucket |
| SAMN42828369 | EUP2136 | Market | Commercial | Carcass |
| SAMN42828370 | EUP2142 | Market | Commercial | Carcass |
| SAMN42828371 | EUP2143 | Market | Commercial | Rinse Bucket |
| SAMN42828372 | EUP2144 | Market | Commercial | Carcass |
| SAMN42828373 | EUP2146 | Market | Commercial | Carcass |
| SAMN42828374 | EUP2148 | Market | Commercial | Carcass |
| SAMN42828375 | EUP2149 | Market | Commercial | Rinse Bucket |
| SAMN42828376 | EUP2150 | Market | Commercial | Carcass |
| SAMN42828377 | EUP2151 | Market | Commercial | Rinse Bucket |
| SAMN42828378 | EUP2152 | Market | Commercial | Carcass |
| SAMN42828379 | EUP2153 | Market | Commercial | Rinse Bucket |
| SAMN42828380 | EUP2160 | Market | Commercial | Carcass |
| SAMN42828381 | EUP2161 | Market | Commercial | Rinse Bucket |
| SAMN42828382 | EUP2162 | Market | Commercial | Carcass |
| SAMN42828383 | EUP2164 | Market | Commercial | Carcass |
| SAMN42828384 | EUP2165 | Market | Commercial | Rinse Bucket |
| SAMN42828385 | EUP2166 | Market | Commercial | Carcass |
| SAMN42828386 | EUP2167 | Market | Commercial | Rinse Bucket |
| SAMN42828387 | EUP2168 | Market | Commercial | Carcass |
| SAMN42828388 | EUP2169 | Market | Commercial | Rinse Bucket |
| SAMN42828389 | EUP2170 | Market | Commercial | Carcass |

Table S2. Metadata for all samples available for sequencing, including those with insufficient DNA for sequencing

| Sample Characteristic | Sequenced (Sufficient DNA) | Not Sequenced  (Insufficient DNA) |
| --- | --- | --- |
| Breed |  |  |
| Commercial | 80 | 243 |
| Local | 21 | 32 |
| Sample Type |  |  |
| Fecal | 26 | 94 |
| Carcass | 49 | 86 |
| Rinse Bucket | 26 | 43 |
| Location |  |  |
| Households (19) | 7 | 13 |
| Farms (26) | 4 | 29 |
| Markets (3) | 90 | 197 |
| Depots (2) | 0 | 4 |
| Supermarkets (6) | 0 | 19 |
| Barracas (4) | 0 | 13 |

Table S3: Antimicrobial Resistance Genes Ranking in the First Quartile of Risk Score from Zhang et al. 2022

| **ARG Name** | **ARG Class** |
| --- | --- |
| APH(6)-Id | aminoglycoside antibiotic |
| APH(3'')-Ib | aminoglycoside antibiotic |
| AAC(6')-Ie-APH(2'')-Ia | aminoglycoside antibiotic |
| acrD | aminoglycoside antibiotic |
| ANT(2'')-Ia | aminoglycoside antibiotic |
| APH(3')-IIIa | aminoglycoside antibiotic |
| kdpE | aminoglycoside antibiotic |
| APH(3')-Ia | aminoglycoside antibiotic |
| ANT(3'')-IIa | aminoglycoside antibiotic |
| AAC(3)-IId | aminoglycoside antibiotic |
| aad(6) | aminoglycoside antibiotic |
| AAC(6')-Ib7 | aminoglycoside antibiotic |
| aadA5 | aminoglycoside antibiotic |
| ANT(4')-Ib | aminoglycoside antibiotic |
| ANT(6)-Ia | aminoglycoside antibiotic |
| CfxA2 | Beta-lactams |
| CfxA3 | Beta-lactams |
| mecA | Beta-lactams |
| TEM-1 | Beta-lactams |
| mecR1 | Beta-lactams |
| CfxA5 | Beta-lactams |
| CfxA4 | Beta-lactams |
| mecI | Beta-lactams |
| OXA-1 | Beta-lactams |
| TEM-95 | Beta-lactams |
| TEM-135 | Beta-lactams |
| TEM-206 | Beta-lactams |
| TEM-214 | Beta-lactams |
| TEM-112 | Beta-lactams |
| CTX-M-15 | Beta-lactams |
| TEM-163 | Beta-lactams |
| FOX-5 | Beta-lactams |
| TEM-196 | Beta-lactams |
| emrR | fluoroquinolone antibiotic |
| emrA | fluoroquinolone antibiotic |
| emrB | fluoroquinolone antibiotic |
| mdtH | fluoroquinolone antibiotic |
| qacA | fluoroquinolone antibiotic |
| qacB | fluoroquinolone antibiotic |
| mdtK | fluoroquinolone antibiotic |
| patB | fluoroquinolone antibiotic |
| patA | fluoroquinolone antibiotic |
| QnrS1 | fluoroquinolone antibiotic |
| mdtG | fosfomycin |
| ErmB | MLS |
| ErmF | MLS |
| mphA | MLS |
| mphC | MLS |
| ErmX | MLS |
| mphB | MLS |
| macB | MLS |
| ErmC | MLS |
| Mef(En2) | MLS |
| RlmA(II) | MLS |
| ErmT | MLS |
| mphE | MLS |
| ErmA | MLS |
| acrB | Multidrug |
| marA | Multidrug |
| H-NS | Multidrug |
| acrF | Multidrug |
| evgS | Multidrug |
| acrE | Multidrug |
| acrS | Multidrug |
| evgA | Multidrug |
| CRP | Multidrug |
| mdtE | Multidrug |
| mdtF | Multidrug |
| tolC | Multidrug |
| gadX | Multidrug |
| gadW | Multidrug |
| mdtM | Multidrug |
| mgrA | Multidrug |
| msrA | Multidrug |
| oqxB | Multidrug |
| efrA | Multidrug |
| mel | Multidrug |
| oqxA | Multidrug |
| MexB | Multidrug |
| cpxA | Multidrug |
| baeR | Multidrug |
| baeS | Multidrug |
| lsaA | Multidrug |
| mexY | Multidrug |
| MexD | Multidrug |
| sdiA | Multidrug |
| OprM | Multidrug |
| mtrD | Multidrug |
| efrB | Multidrug |
| adeF | Multidrug |
| mexI | Multidrug |
| mexK | Multidrug |
| MexA | Multidrug |
| smeE | Multidrug |
| smeB | Multidrug |
| OpmB | Multidrug |
| AxyY | Multidrug |
| MexF | Multidrug |
| lsaE | Multidrug |
| ramA | Multidrug |
| mexW | Multidrug |
| smeD | Multidrug |
| AxyX | Multidrug |
| vgaA | Multidrug |
| mdsB | Multidrug |
| MexE | Multidrug |
| mtrC | Multidrug |
| norA | Multidrug |
| msrC | Multidrug |
| smeF | Multidrug |
| mtrA | Multidrug |
| arlR | Multidrug |
| MexC | Multidrug |
| mexQ | Multidrug |
| arlS | Multidrug |
| PmpM | Multidrug |
| efmA | Multidrug |
| OprZ | Multidrug |
| smeS | Multidrug |
| poxtA | Multidrug |
| ceoB | Multidrug |
| mexH | Multidrug |
| lsaC | Multidrug |
| LAP-2 | Multidrug |
| adeJ | Multidrug |
| ugd | peptide antibiotic |
| eptA | peptide antibiotic |
| pmrF | peptide antibiotic |
| bacA | peptide antibiotic |
| yojI | peptide antibiotic |
| catI | phenicol antibiotic |
| sul1 | sulfonamide antibiotic |
| sul2 | sulfonamide antibiotic |
| tetO | tetracycline antibiotic |
| tetM | tetracycline antibiotic |
| emrY | tetracycline antibiotic |
| tetQ | tetracycline antibiotic |
| tet(W/N/W) | tetracycline antibiotic |
| emrK | tetracycline antibiotic |
| tet(B) | tetracycline antibiotic |
| tet(A) | tetracycline antibiotic |
| tet(40) | tetracycline antibiotic |
| tetX | tetracycline antibiotic |
| Tet(X4) | tetracycline antibiotic |
| tet(K) | tetracycline antibiotic |
| Tet(X3) | tetracycline antibiotic |
| tet(L) | tetracycline antibiotic |
| tetW | tetracycline antibiotic |
| tet(C) | tetracycline antibiotic |
| tet(D) | tetracycline antibiotic |
| tetA(P) | tetracycline antibiotic |
| tet32 | tetracycline antibiotic |


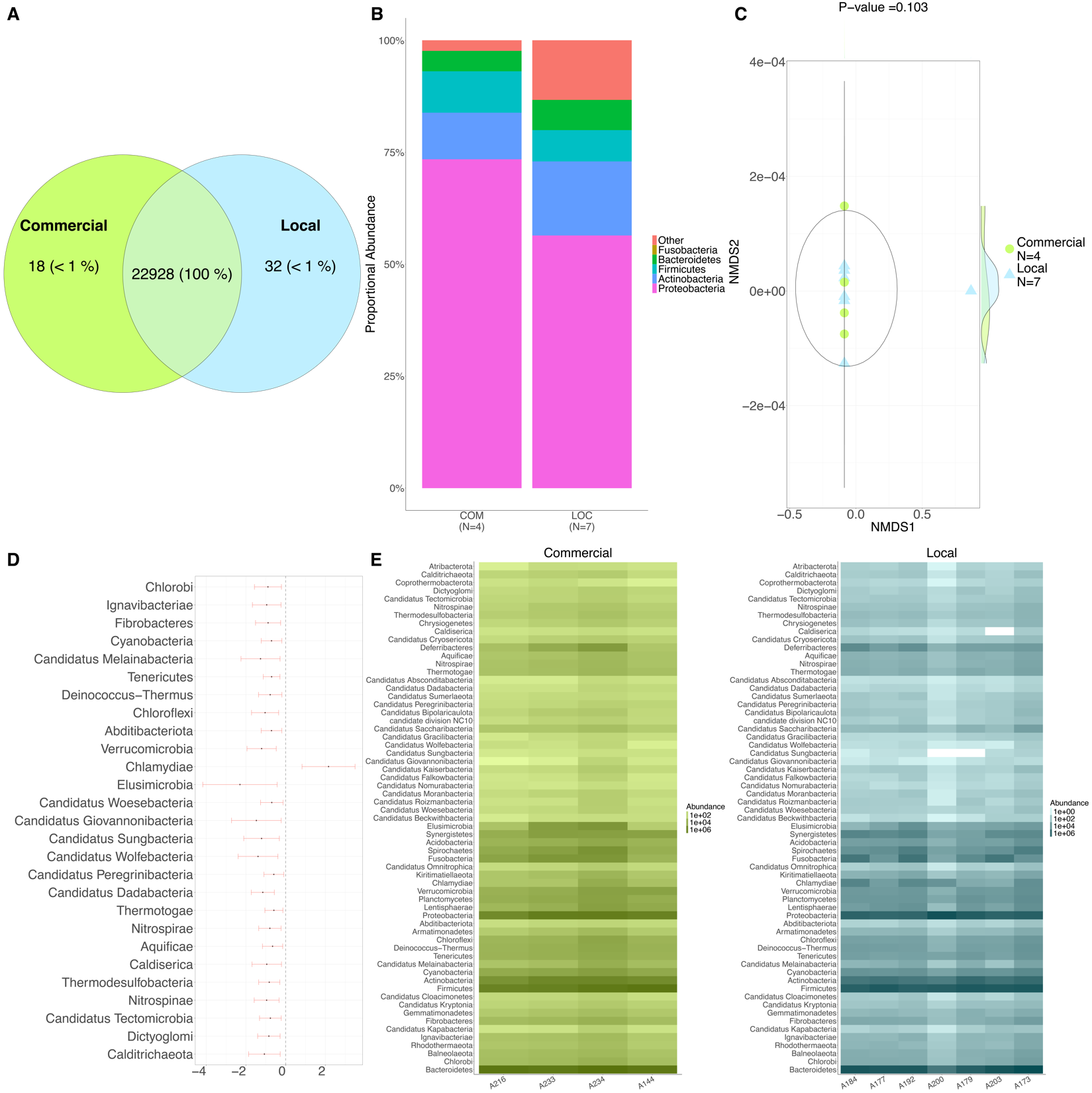


**Figure S1. Summary of bacteria detected in fecal metagenomes from poultry production sites (farms or households).** A) Venn diagram depicting absolute number of taxa detected in commercial versus local chickens. B) Stacked bar plot depicting proportional abundance of taxa detected in commercial versus local chickens. C) Non-metric multidimensional scaled plot of Bray Curtis distances depicting bacterial community composition. D) Differentially abundant taxa among local versus commercial (reference) chickens. E) Heatmaps of log10 transformed taxa abundance.


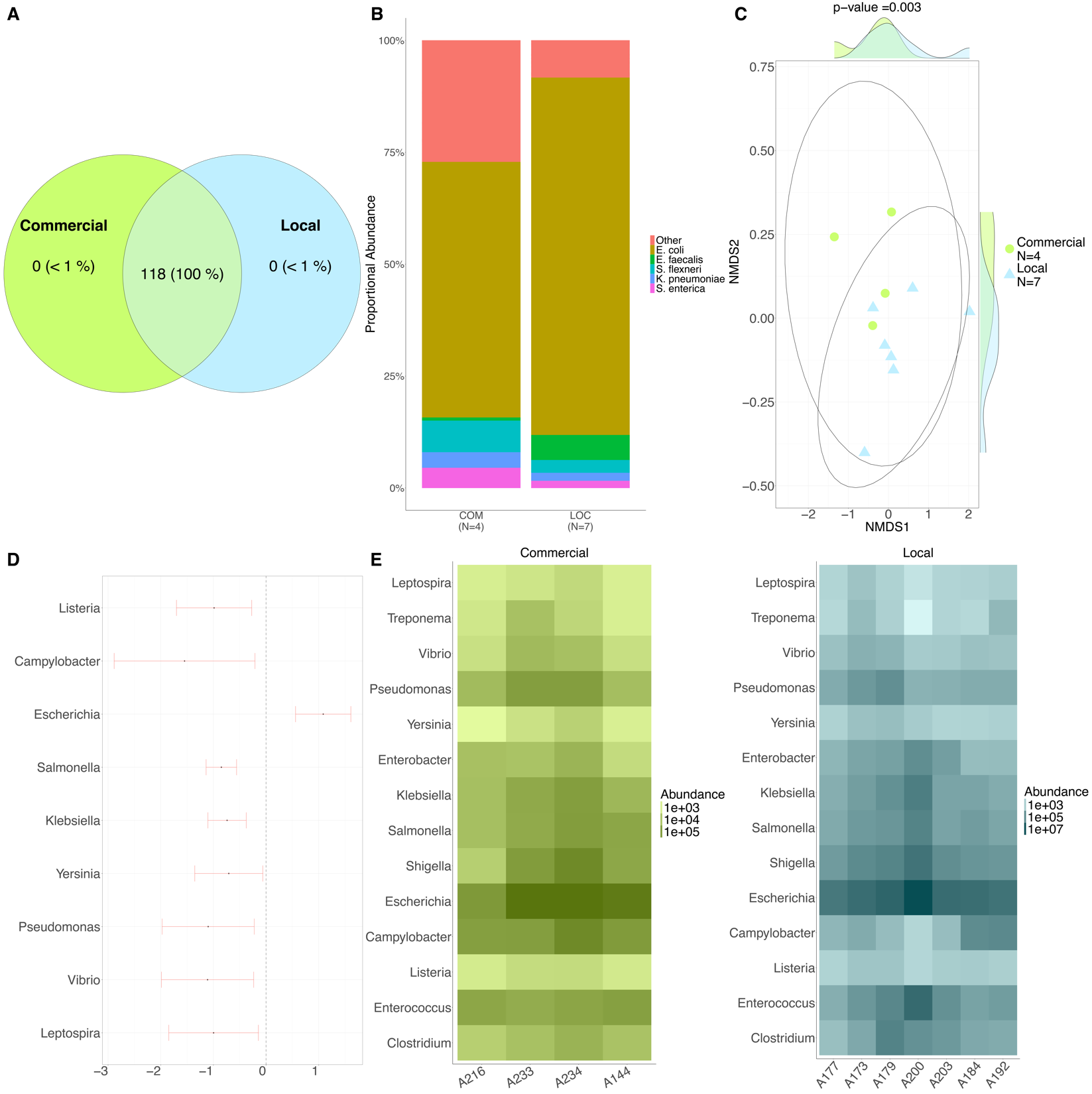


**Figure S2. Summary of potential human pathogens detected in fecal metagenomes from poultry production sites (farms or households).** A) Venn diagram depicting absolute number of pathogens detected in commercial versus local chickens. B) Stacked bar plot depicting proportional abundance of pathogens detected in commercial versus local chickens. C) Non-metric multidimensional scaled plot of Bray Curtis distances depicting bacterial pathogen community composition. D) Differentially abundant pathogens among local versus commercial (reference) chickens. E) Heatmaps of log10 transformed pathogen abundance.


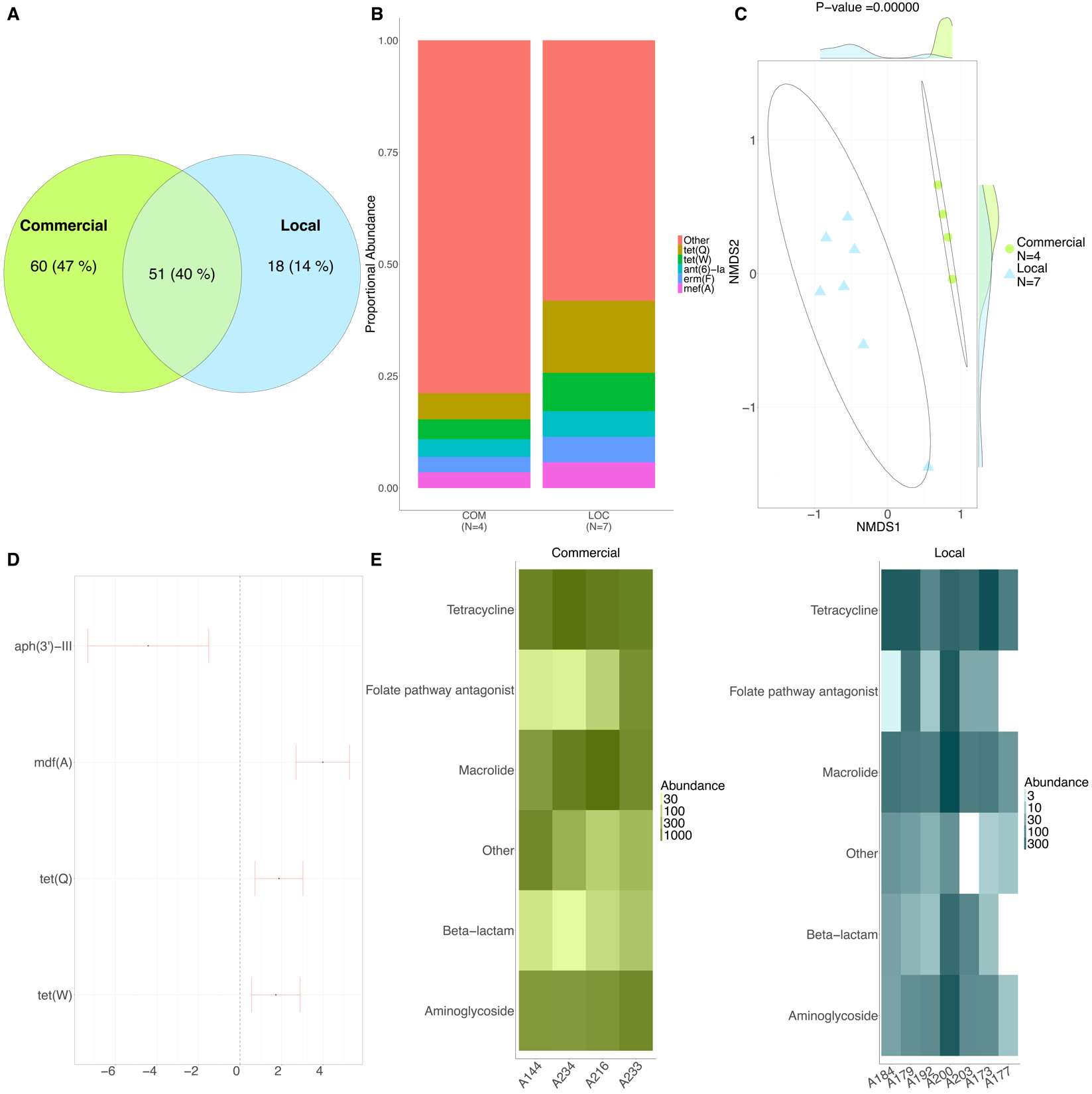


**Figure S3. Summary of ARGs detected in fecal metagenomes from poultry production sites (farms or households).** A) Venn diagram depicting absolute number of ARGs detected in commercial versus local chickens. B) Stacked bar plot depicting proportional abundance of ARGs detected in commercial versus local chickens. C) Non-metric multidimensional scaled plot of Bray Curtis distances depicting ARG composition. D) Differentially abundant ARGs among local versus commercial (reference) chickens. E) Heatmaps of log10 transformed ARG abundance.


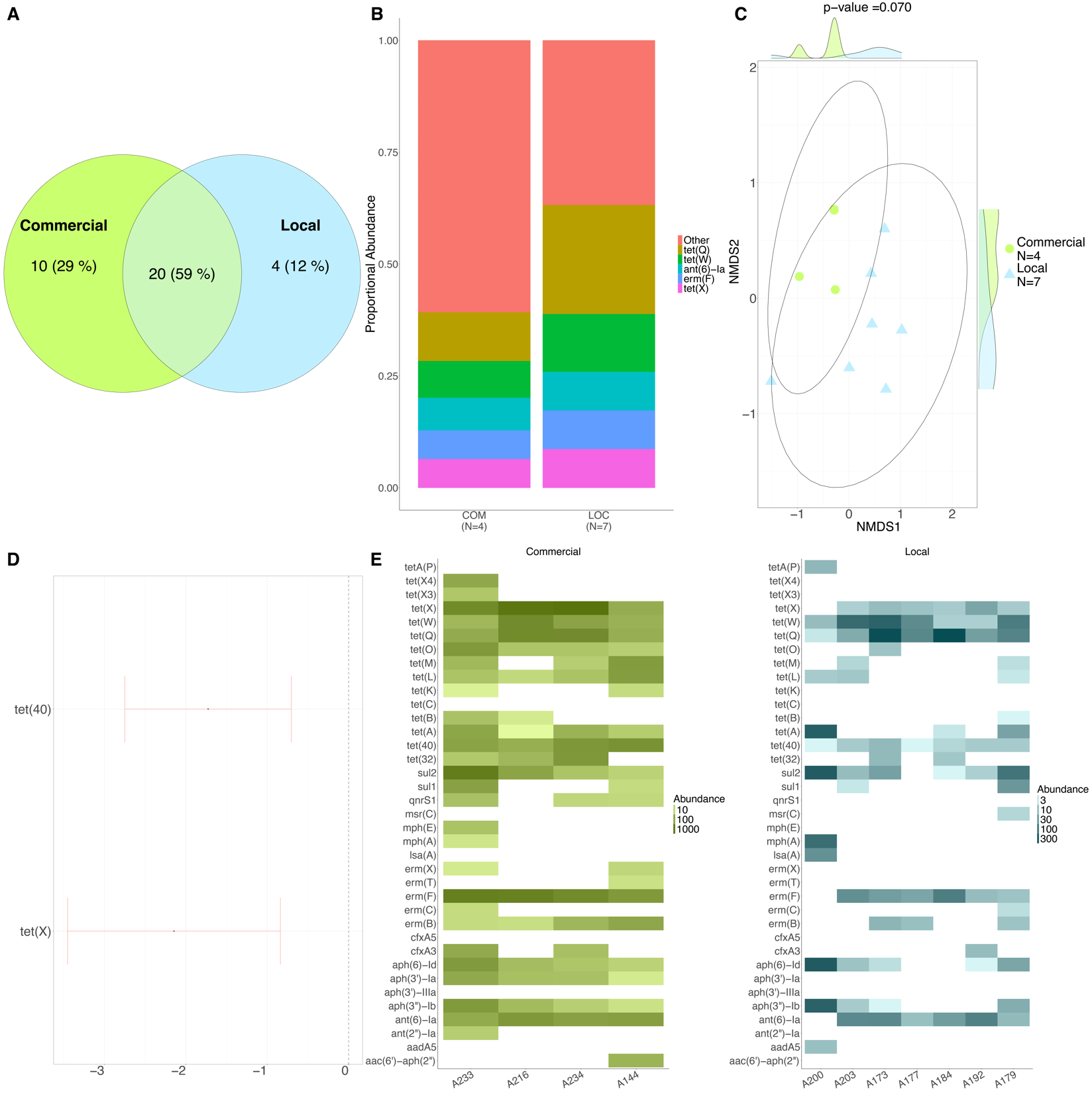


**Figure S4. Summary of high-risk ARGs (HR-ARGs) detected in fecal metagenomes from poultry production sites (farms or households).** A) Venn diagram depicting absolute number of HR-ARGs detected in commercial versus local chickens. B) Stacked bar plot depicting proportional abundance of HR-ARGs detected in commercial versus local chickens. C) Non-metric multidimensional scaled plot of Bray Curtis distances depicting HR-ARG composition. D) Differentially abundant HR-ARGs among local versus commercial (reference) chickens. E) Heatmaps of log10 transformed HR-ARG abundance.


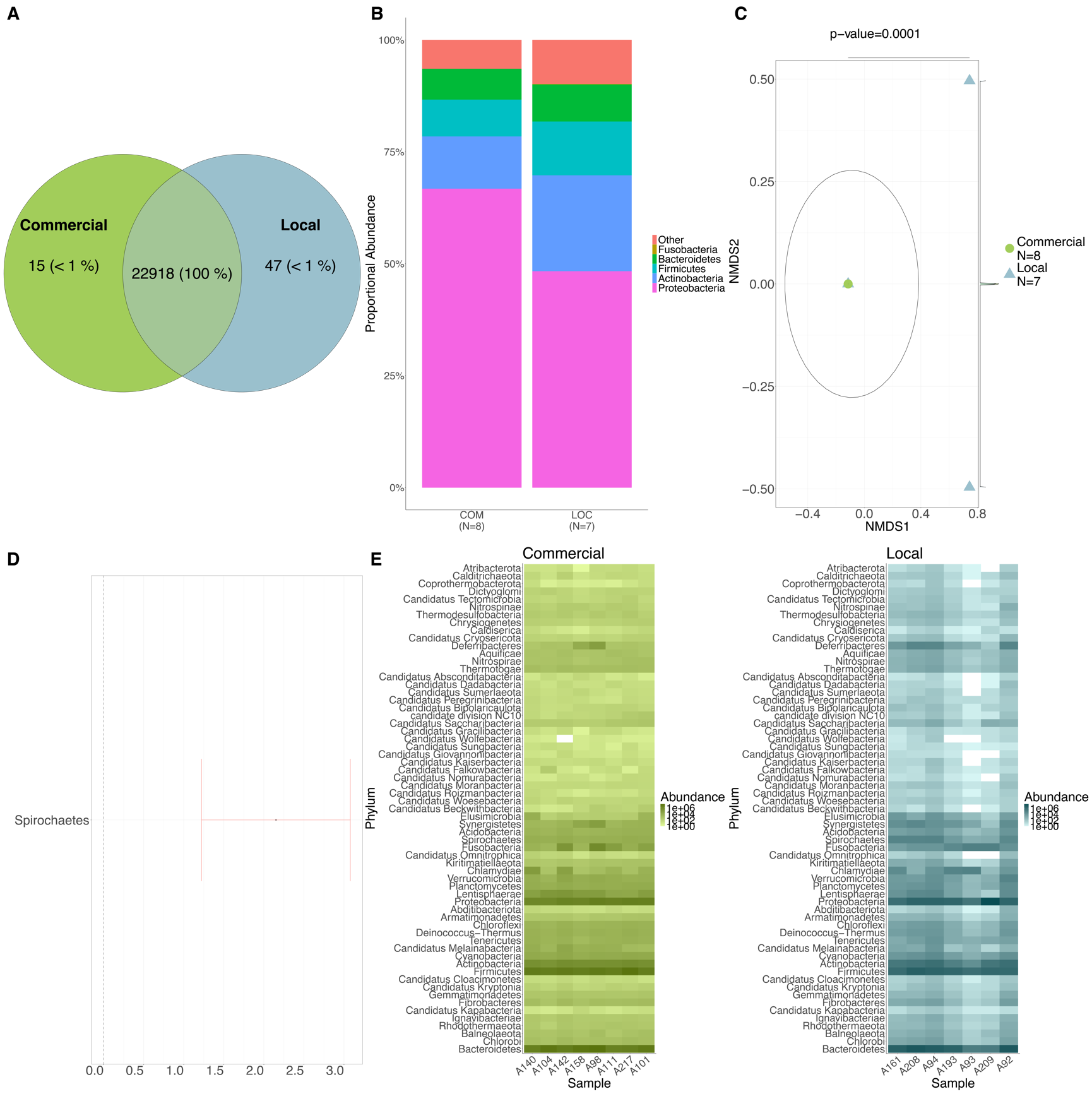
**Figure S5. Summary of bacteria detected in fecal metagenomes from market sites.**

A) Venn diagram depicting absolute number of taxa detected in commercial versus local chickens. B) Stacked bar plot depicting proportional abundance of taxa detected in commercial versus local chickens. C) Non-metric multidimensional scaled plot of Bray Curtis distances depicting bacterial community composition. D) Differentially abundant taxa among local versus commercial (reference) chickens. E) Heatmaps of log10 transformed taxa abundance.


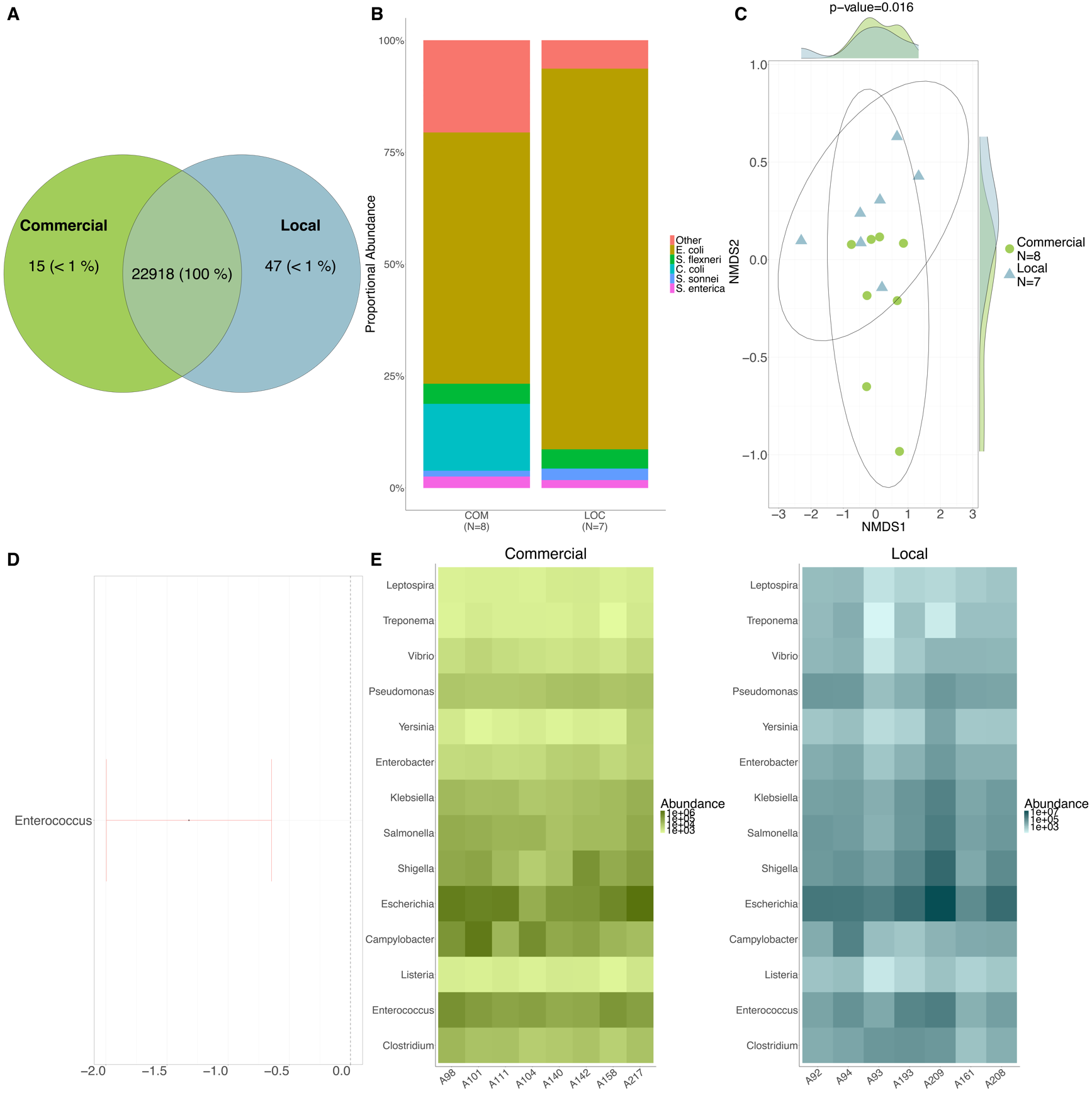


**Figure S6. Summary of potential human pathogens detected in fecal metagenomes from market sites.** A) Venn diagram depicting absolute number of pathogens detected in commercial versus local chickens. B) Stacked bar plot depicting proportional abundance of pathogens detected in commercial versus local chickens. C) Non-metric multidimensional scaled plot of Bray Curtis distances depicting bacterial pathogen community composition. D) Differentially abundant pathogens among local versus commercial (reference) chickens. E) Heatmaps of log10 transformed pathogen abundance.


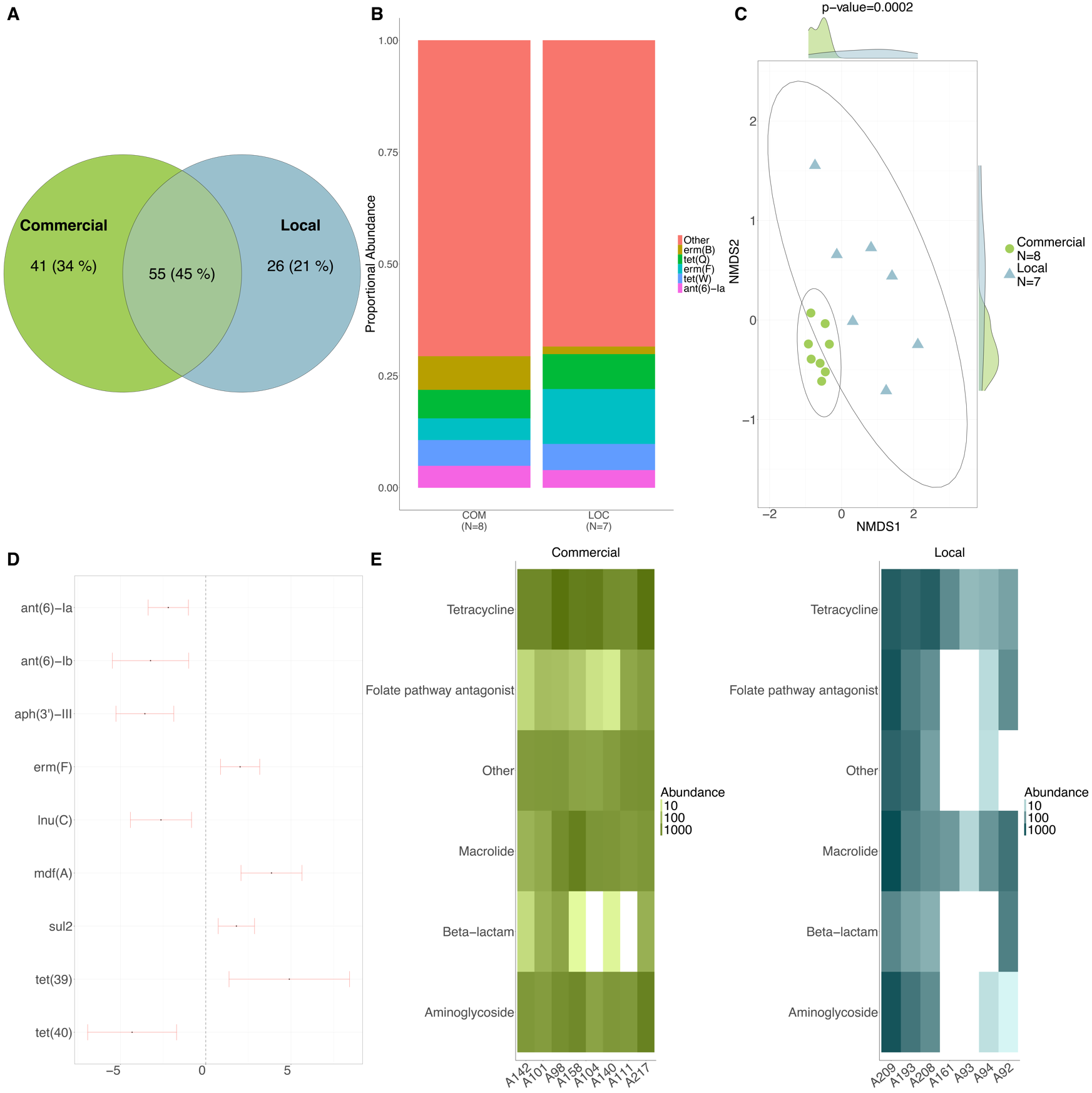
**Figure S7. Summary of ARGs detected in fecal metagenomes from market sites.** A) Venn diagram depicting absolute number of ARGs detected in commercial versus local chickens. B) Stacked bar plot depicting proportional abundance of ARGs detected in commercial versus local chickens. C) Non-metric multidimensional scaled plot of Bray Curtis distances depicting ARG composition. D) Differentially abundant ARGs among local versus commercial (reference) chickens. E) Heatmaps of log10 transformed ARG abundance.


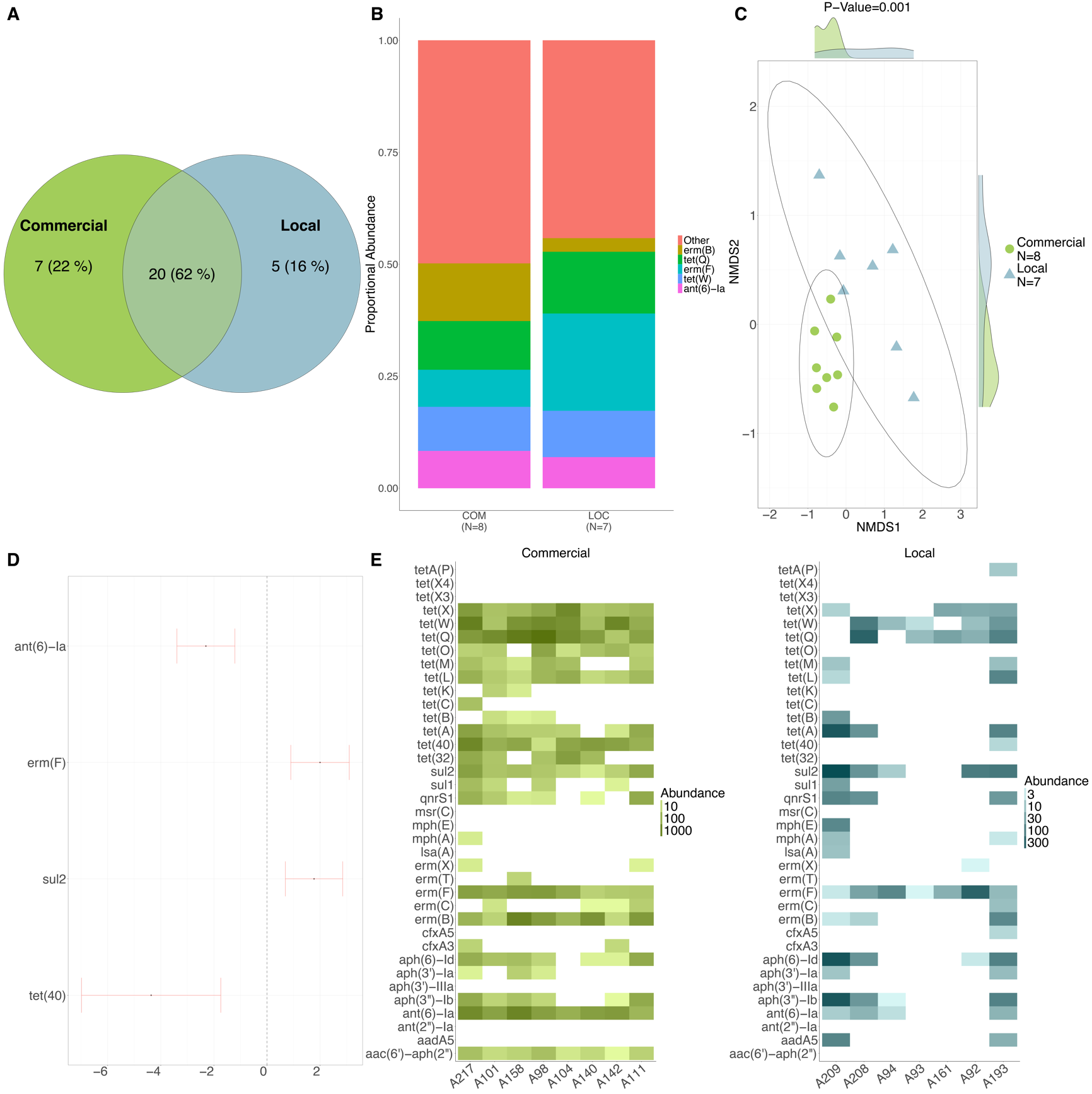
**Figure S8. Summary of high-risk ARGs (HR-ARGs) detected in fecal metagenomes from market sites.** A) Venn diagram depicting absolute number of HR-ARGs detected in commercial versus local chickens. B) Stacked bar plot depicting proportional abundance of HR-ARGs detected in commercial versus local chickens. C) Non-metric multidimensional scaled plot of Bray Curtis distances depicting HR-ARG composition. D) Differentially abundant HR-ARGs among local versus commercial (reference) chickens. E) Heatmaps of log10 transformed HR-ARG abundance.


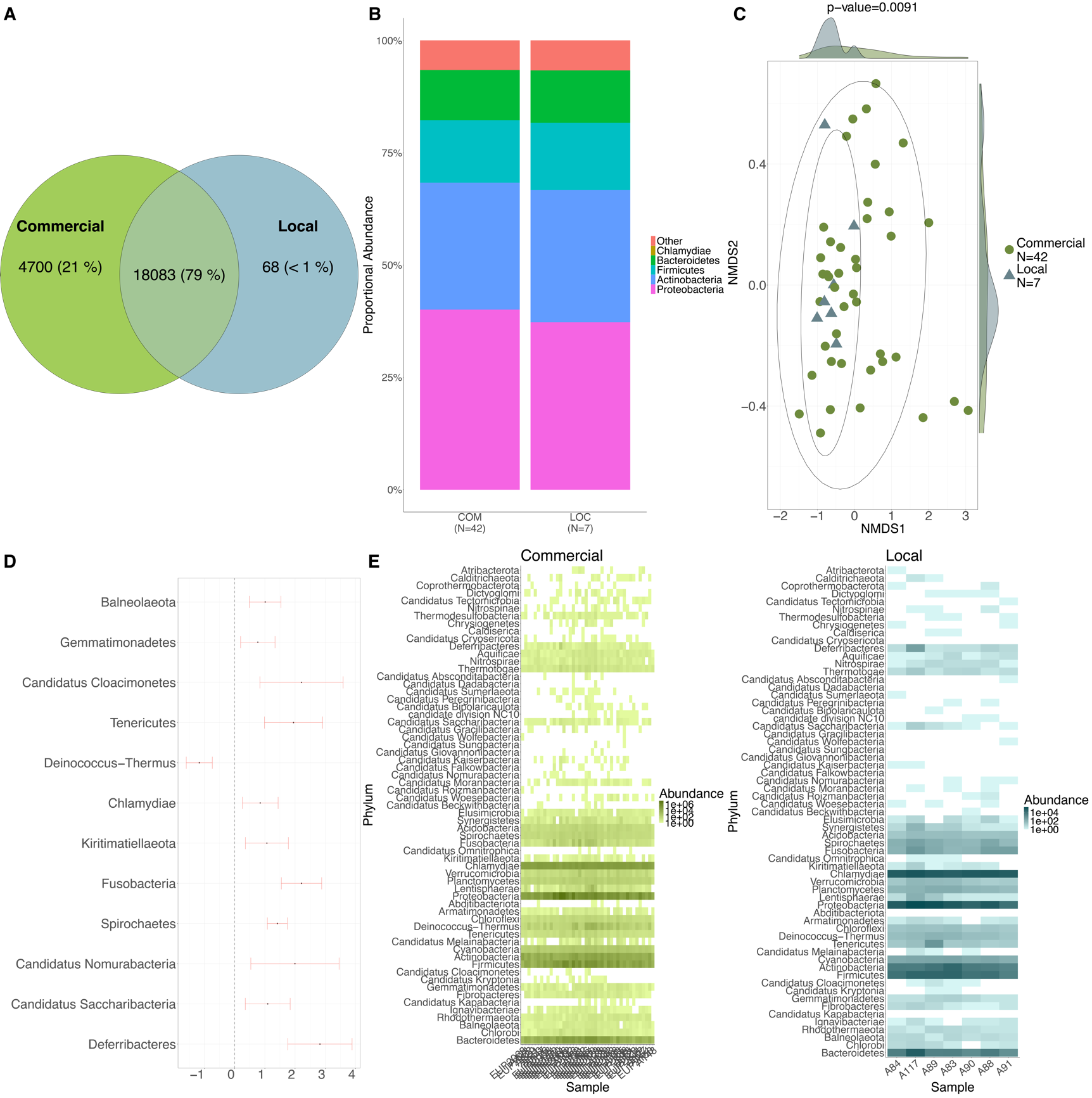
**Figure S9. Summary of bacteria detected in poultry carcass metagenomes from market sites.**

A) Venn diagram depicting absolute number of taxa detected in commercial versus local chickens. B) Stacked bar plot depicting proportional abundance of taxa detected in commercial versus local chickens. C) Non-metric multidimensional scaled plot of Bray Curtis distances depicting bacterial community composition. D) Differentially abundant taxa among local versus commercial (reference) chickens. E) Heatmaps of log10 transformed taxa abundance.


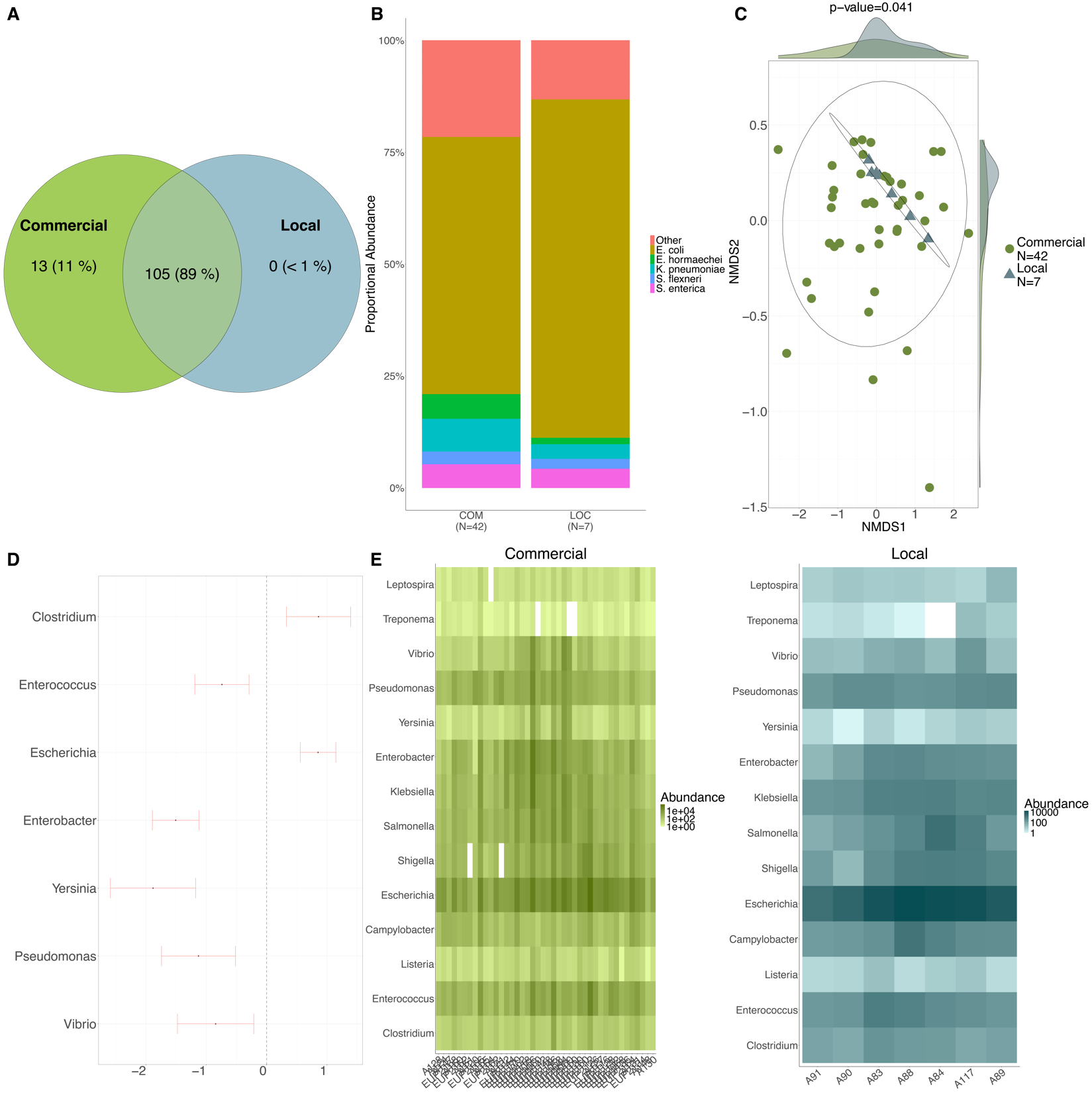
**Figure S10. Summary of potential human pathogens detected in poultry carcass metagenomes from market sites.** A) Venn diagram depicting absolute number of pathogens detected in commercial versus local chickens. B) Stacked bar plot depicting proportional abundance of pathogens detected in commercial versus local chickens. C) Non-metric multidimensional scaled plot of Bray Curtis distances depicting bacterial pathogen community composition. D) Differentially abundant pathogens among local versus commercial (reference) chickens. E) Heatmaps of log10 transformed pathogen abundance.


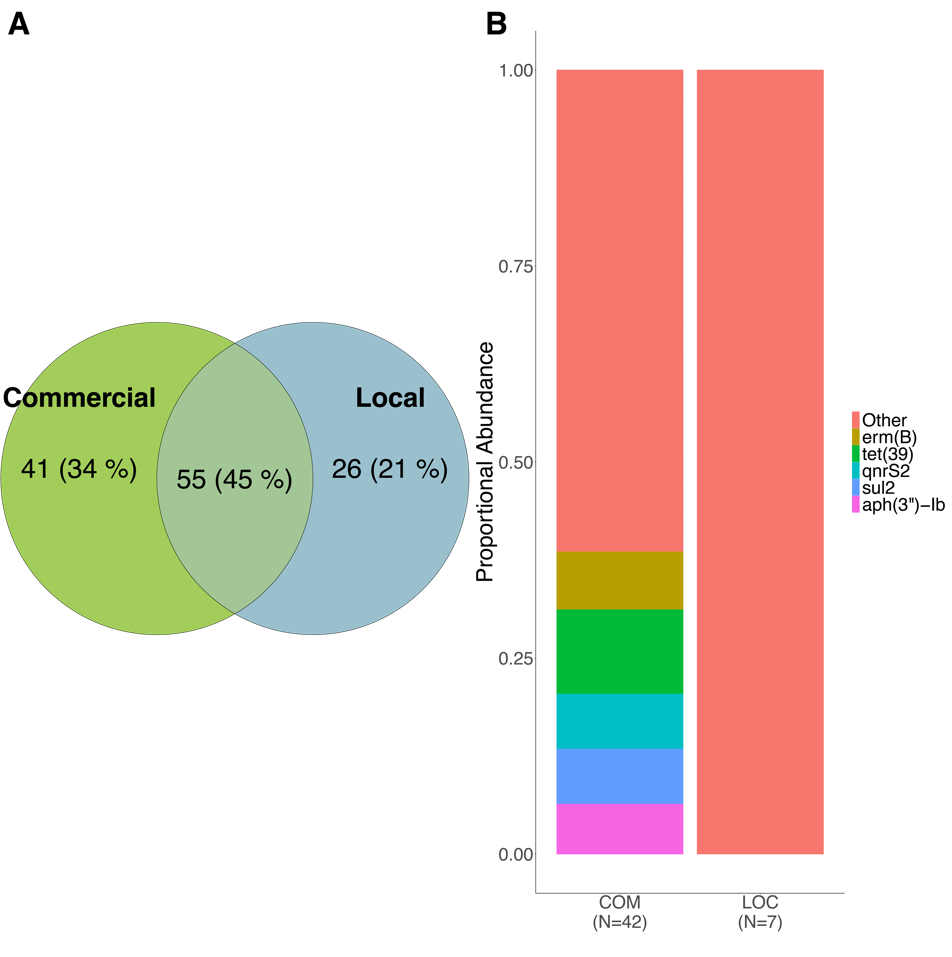


**Figure S11. Summary of ARGs detected in poultry carcass metagenomes from market sites.** A) Venn diagram depicting absolute number of ARGs detected in commercial versus local chickens. B) Stacked bar plot depicting proportional abundance of ARGs detected in commercial versus local chickens.
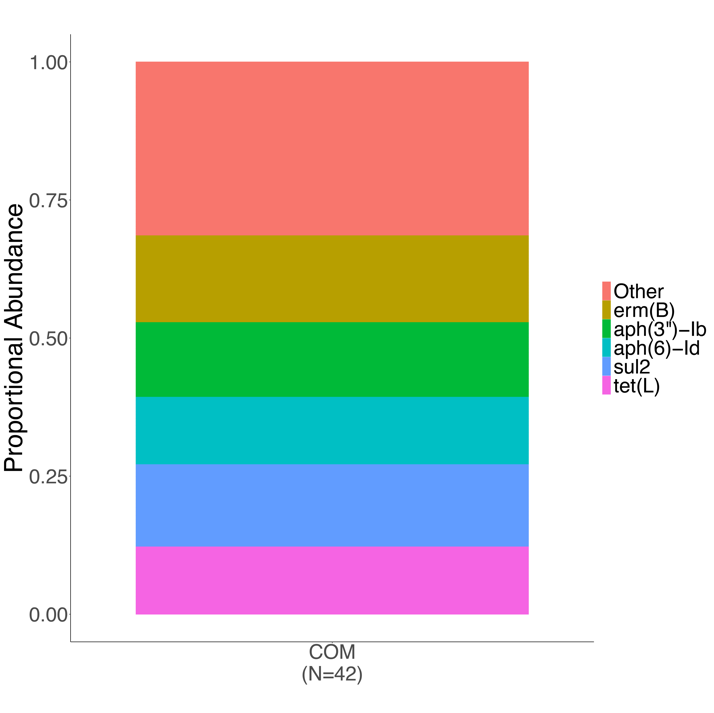


**Figure S12. Stacked bar plot depicting proportional abundance of high-risk ARGs (HR-ARGs) detected in commercial carcass metagenomes from market sites.**
